# Comparative analysis of HKTs in six poplar species and functional characterization of PyHKTs in stress-affected tissues

**DOI:** 10.1186/s12864-025-11203-x

**Published:** 2025-01-07

**Authors:** Xiaojiao Liu, Lincui Shi, Hezi Bai, Jing Wang, Anmin Yu, Aizhong Liu, Ping Li

**Affiliations:** 1 Key Laboratory for Forest Resource Conservation and Utilization in the Southwest Mountains of China (Ministry of Education), College of Forestry, Southwest Forestry University, Kunming, China; 2 Yunnan Provincial Key Laboratory for Conservation and Utilization of In-forest Resource, Southwest Forestry University, Yunnan Kunming, China

**Keywords:** HKT, *Populus*, Evolution, Expression, Stress

## Abstract

Plant HKTs (High-affinity K^+^ transporters) are essential transporters for ion transport and homeostasis and play crucial roles in plant growth and stress responses. However, the evolution of HKTs in *Populus* species and their functions require further investigation. In this study, we identified 16 HKTs from six *Populus* species. All poplar HKTs were classified as Class I HKTs because of their physiological relationships and the conservation of amino acids in key structures, which aligns with their conserved evolutionary coding sequences. The analysis of the protein domains, motifs and gene structures of 16 poplar HKTs revealed consistent conservation, with the exception of two members. The number of homologs and their chromosome locations indicated the differentiation of HKTs during poplar evolution and adaptation. Poplar HKTs can be classified into two subgroups on the basis of their physiological relationships and distinct protein structures. Gene expression pattern analysis revealed that poplar HKTs presented relatively high expression levels in roots and stems under salt stress. Furthermore, cis-element analysis and protein interaction predictions provide insights into the functions of HKTs under salt stress through the activation of ion transporters, proline content, and ATPases regulated by hormonal signals and MYB transcription factors. In conclusion, our research established a theoretical framework for investigating the evolutionary relationships and functional roles of HKTs in *Populus* species and offered valuable insights into the functions and underlying mechanisms of poplar HKTs in specific tissues under various stress conditions.

**Supplementary Information:**

The online version contains supplementary material available at 10.1186/s12864-025-11203-x.

## Introduction

The increasing soil salinity caused by climate change and irrigation farming is a serious threat to agriculture worldwide [1]. The increasing concentration of Na^+^ in the soil inhibits the water uptake capability of plant roots [2]. The osmotic stress caused by an increase in Na^+^ can be alleviated through cation transport within plants [3]. High-affinity K^+^ transporters (HKTs) belong to the Ktr/Trk/HKT transporter family, which are K^+^ channels generally located in the cell membrane that are involved in ion transport and cell homeostasis [4]. HKTs are channel proteins involved in K^+^ uptake and Na^+^-K^+^ homeostasis, which are crucial for plant salt tolerance and Na^+^ uptake under K^+^ shortage conditions [5]. A class of HKT transporters found in both dicot and monocot plants can prevent Na^+^ accumulation in shoots by mediating Na^+^ exclusion from xylem vessels under salt stress [1].

Plant HKT proteins contain four loops in a polypeptide, which are homologous to K^+^ channels [6]. Glycine residues, especially those in P-loop A, are important for the selective filter function of HKT proteins [6]. The conserved Arg and Gly at the beginning of the transmembrane segment are also important for the cation transport activity of HKT [4]. HKTs from different plants can be divided into two classes according to their phylogenetic relationships and cation transport properties [7, 8]. Class I HKT transporters, such as *Arabidopsis* HKT1, show greater Na^+^ affinity [9]. Class II HKTs, such as TaHKT2;1, exhibit increased K^+^ penetration [10].

Different HKTs can be obtained during the natural growth of *Mocho de Espiga Branca*, where a single nucleotide substitution of TaHKT1;5-D inhibited the release of Na^+^ in shoots and decreased salt tolerance [11]. The expression of SbHKT1;4 (a HKT of *Sorghum bicolor*) was strongly upregulated under salt stress, which could mediate K^+^ uptake under Na^+^ stress and improve plant salt tolerance [12]. HKTs from both *Triticum monoccocum* and *Triticum aestivum* improve Na^+^ exclusion, which leads to improved plant salt tolerance [13]. TaHKT1;5-D (a HKT of bread wheat) is important for the salinity tolerance of wheat because it restricts the transport of Na^+^ from roots to leaves [14].

The expression and function of HKTs are different in different plants. The two HKT homologs in *Thellungiella salsuginea* demonstrated functional differentiation; TsHKT1;1 exhibited low abundance, while TsHKT1;2 was strongly upregulated in response to salt stress [15]. Rice HAKs also exhibited functional differentiation when heterogeneously expressed in *Escherichia coli*, and both OsHAKs rescued bacterial growth by increasing the K^+^ content. However, OsHAK5 functions as a Na^+^-insensitive K^+^ transporter, whereas OsHAK2 is sensitive to extracellular Na^+^ and inhibits K^+^ transport [16]. Two HKT transporters of *Triticum monococcum* presented similar expression patterns in the leaves but presented different expression profiles and responses in the roots and under salt stress [17]. Bioinformatics analysis and activity verification of the TmHKT1;4-A2 promoter revealed an enrichment of cis-elements associated with abiotic stress and phytohormones, which accounts for the response of HKTs to salt, osmotic stress, heavy metals, oxidative stress, and hormone stimuli [18]. The variability in K^+^ affinity among different HKTs contributed to enhanced Na^+^ exclusion in shoots and improved salt tolerance in plants [19].

Poplar, a widespread forest tree, has undergone differentiation in terms of morphological, physiological and genetic characteristics during the evolution and adaptation of different species [20]. On the other hand, poplar is a noteworthy tree species not only for its genetic, taxonomic, and evolutionary investigation but also for its rapid growth and economic and ecological relevance [21–23]. Despite the function of HKTs being well established in many plants, their distribution, characteristics, and function in poplar remain unclear. In this study, we identified 16 HKTs in six *Populus* species, all of which were classified as Class I HKTs on the basis of their physiological relationships and conserved amino acids, in accordance with the evolutionary rate of protein-coding genes (Ka/Ks ratio). On the basis of their physiological relationships, the 16 poplar HKTs can be classified into two subgroups. The varying number and chromosomal locations of these HKTs contributed to the evolutionary differentiation of poplar HKTs. The protein domains, motifs, and gene structures indicated the conservation of poplar HKTs; however, divergence also existed among HKT homologs. The expression patterns demonstrated that poplar HKTs were tissue specific and stress responsive, in accordance with the predicted regulatory factors and proteins. Our findings provide a theoretical foundation for subsequent studies on the potential functions of HKTs in *Populus* species.

## Materials and methods

### HKT protein acquisition and bioinformatics analysis

*Populus* HKT proteins were obtained via the local BLAST search tool (blast-2.14.1+, https://blast.ncbi.nlm.nih.gov/Blast.cgi) with an E_value threshold of < 0.05, employing the reported *Arabidopsis* HKT protein (AtHKT1, AAF68393.1) as a query against the poplar genome database [8]. Genome data for six species of poplar were obtained from NCBI (https://www.ncbi.nlm.nih.gov/datasets/genome/) and the National Genomics Data Center (https://bigd.big.ac.cn/bioproject) [20]. A physiological tree of candidate poplar HKTs, AtHKT1, and wheat TaHKT2;1 was constructed via MEGA (version 11.0.9) with amino acid sequences. The maximum likelihood method was employed with 1000 bootstrap replications and a partial deletion cutoff of 80% site coverage [24].

Smart (http://smart.embl.de/) [25] and Batch CD Search (https://www.ncbi.nlm.nih.gov/Structure/cdd/wrpsb.cgi) [26] were utilized to identify the conserved domains of candidate poplar HKTs. Additionally, the protein sequences were analyzed via the ExPASy website (https://www.expasy.org/) to retrieve information, including the number of amino acids, molecular weight (MW), isoelectric point (pI), aliphatic index, and hydrophilicity index. The subcellular localization of candidate poplar HKTs was predicted via WoLF PSORT (https://wolfpsort.hgc.jp/).

### Bioinformatic analysis of poplar HKTs

The 3D reconstruction map of PyHKTs was constructed via the SWISS-MODEL Workspace of ExPASy (https://swissmodel.expasy.org) [27]. The characteristic motifs were analyzed via MEME (Multiple Em for Motif Elicitation, Version 5.5.5, https://meme-suite.org/meme/tools/meme) with a threshold of 10 motifs. The evolutionary trees of the poplar HKTs were constructed via MEGA 11 (version 11.0.9) [24]. Protein motifs, domains and gene structures were illustrated via TBtools-II (Toolbox for Biologists v2.110) [27].

Cis-elements were identified using PlantCARE website (http://bioinformatics.psb.ugent.be/webtools/plantcare/html/) and illustrated via TB tools with 2000 bp DNA sequences preceding the transcription start sites (ATGs) of the coding genes of poplar HKTs. Chromosomal localization maps of the *HKT* genes were generated via gene location visualization via the GTF/GFF package of TBtools software with the genome annotation data of poplar HKTs.

### Plant materials and treatment

The experimental materials were two-month-old poplar (*Populus yunnanensis*) seedlings cultivated in mixed nutrient pots (humus: quartz sand: perlite at 3:1:1) in the greenhouse of Southwest Forestry University. In this study, healthy *P. yunnanensis* plants were selected for various stress treatments, which included saline treatment (150 mmol/L NaCl for 1 day), abscisic acid (ABA, 50 µmol/mL for 1 day), drought treatment (no water for 2 days), high-temperature treatment (45 °C for 1 day), and D-mannitol treatment (25% mannitol for 2 days). The treatment methods were adapted from the preliminary experimental scheme established by the research group [28]. Following treatment, the leaf samples were wrapped in aluminum foil, labeled, and quickly frozen in liquid nitrogen before being stored in a -80 °C freezer for future use by the research group.

### RNA extraction, reverse transcription and fluorescence quantitative PCR

Approximately 0.1 g of each *P. yunnanensis* sample subjected to different stress treatments was weighed and subsequently ground into a fine powder in a mortar using liquid nitrogen. Total RNA (ribonucleic acid) was extracted in accordance with the protocol provided by the RNAprep Pure Polysaccharide Polyphenol Plant Total Extraction Kit (Tiangen, DP190813). The quality of the RNA was assessed via 1% agarose gel electrophoresis and a NanoDrop Spectrophotometer (Thermo Fisher Scientific). On the basis of the cDNA sequences of the *P. yunnanensis HKT* genes, primers were designed via Primer Premier 5.0 software adhering to the principles of quantitative fluorescent primers (Table S1).

The total RNA of *P. yunnanensis* was reverse-transcribed via the All-in-One First-Strand cDNA Synthesis SuperMix for PCR Kit (AT321, Transgene) following the manufacturer’s instructions. The reverse transcription reaction was conducted at 42 °C for 60 min, followed by 70 °C for 10 min, and the samples were subsequently cooled to 4 °C for storage. The reverse transcription product was utilized in subsequent fluorescence quantitative PCR experiments.

Quantitative fluorescence PCR was conducted via qRT-PCR (Quantitative Reverse Transcription Polymerase Chain Reaction, TransStart^®^ Tip Green qPCR SuperMix (AQ141-01)) with PyEF1 serving as the reference gene. Reactions were performed using a Bio-Rad CFX96 instrument (Bio-Rad) [28].

## Results

### Screening and identification of HKT proteins in six *Populus* species

Using the reported AtHKT1 protein sequence from *Arabidopsis* (AAF68393, AT4G10310) as a reference, a local BLASTP search was conducted on the whole-genome protein sequences of six *Populus* species. Furthermore, an NCBI-CD batch search was employed to identify the characteristic domain (cl30043). Across the six *Populus* species, a total of 16 HKT proteins were identified, as summarized in Table 1. The physicochemical properties of the 16 identified poplar HKT proteins were analyzed, yielding the following insights: the amino acid count ranged from 166 (Poyun34820) to 547 (XP_011017603). The molecular weights (MWs) of these proteins ranged from 18.89012 kDa (Poyun34820) to 61.88973 kDa (XP_011017603.1). The isoelectric points (pIs) of these proteins varied between 8.99 (Podel. 18G152700) to 9.62 (Potri.018G147501), suggesting that the poplar HKT proteins are basic in nature. The aliphatic indices of these proteins ranged from 97.38 (Potri.018G147501.2) to 106.4 (XP_011017603.1). Additionally, the GRAVY values, which all exceeded − 0.5, ranged from − 0.067 (Potri.018G147501.2) to 0.419 (XP_011017603.1), indicating the hydrophobic nature of the poplar HKT proteins. When considering subcellular localization, with most proteins located in the cell membrane, Potri.018G147501 located in the thylakoid membrane, and Poyun34820 in the vacuolar membrane, it is inferred that poplar HKT proteins were likely membrane-embedded and function within these contexts. The acquisition of these physicochemical properties thus laid the groundwork for subsequent bioinformatic analyses of HKT genes within the poplar genome.

**Table 1:** Information on HKT proteins from six poplar tree species

ID	Number of amino acids	Molecular weight (kD)	Theoretical pI	Aliphatic index	Grand average of hydropathicity (GRAVY)	location
XP_011017602.1	521	58.91411	9.03	105.01	0.365	plas: 8
XP_011017603.1	547	61.88973	9.24	106.4	0.419	plas: 9
XP_011017604.1	521	58.88206	9.19	104.63	0.36	plas: 8
XP_011017605.1	521	58.99117	9.11	105.76	0.354	plas: 8
XP_011017751.1	535	59.92073	9.29	103.76	0.351	plas: 9
XP_034906209.1	521	58.94505	9.02	101.84	0.332	plas: 11
XP_034918867.1	535	60.02179	9.41	101.38	0.321	plas: 7
Podel.18G140400	535	60.15903	9.45	101.76	0.296	plas: 7
Podel.18G152700	521	58.88908	8.99	103.7	0.365	plas: 11
KAG6740023.1	473	52.59777	9.16	103.78	0.291	plas: 5
KAG6741040.1	535	60.03582	9.41	101.38	0.321	plas: 7
KAG6741120.1	500	56.53539	9.16	104.94	0.358	plas: 8
Potri.018G132200	535	60.09387	9.45	100.11	0.293	plas: 8
Potri.018G147501	168	19.39676	9.62	97.38	-0.067	chlo: 5
Poyun34820	166	18.89012	9.1	102.05	0.11	vacu: 6
Poyun34689	535	59.97982	9.42	100.65	0.313	plas: 11

MW: Molecular weight; GRAVY: Grand average of hydropathicity

### Physiology analysis of poplar HKTs

On the basis of existing knowledge regarding HKTs, two subgroups are classified according to their physiological relationships and cation transport activities [8]. *Arabidopsis* AtHKT1 is categorized as belonging to Class I, characterized by its first loop region containing serine, which is designated a Na⁺-specific transporter [9]. Conversely, wheat TaHKT2;1 is classified as belonging to Class II, wherein the serine in the Class II loop region is substituted with glycine, thereby rendering it a K⁺-selective transporter [10]. Phylogenetic analysis of known classified HKT proteins from *Arabidopsis* and wheat revealed that wheat HKT proteins formed a separate branch, whereas the 16 identified poplar HKT proteins clustered with *Arabidopsis* AtHKT1 within the same evolutionary branch (Figure S1). This preliminary analysis revealed that the identified HKT proteins in poplars were classified as Class I.

### Sequence and structural analysis of poplar HKTs

**Fig. 1:**
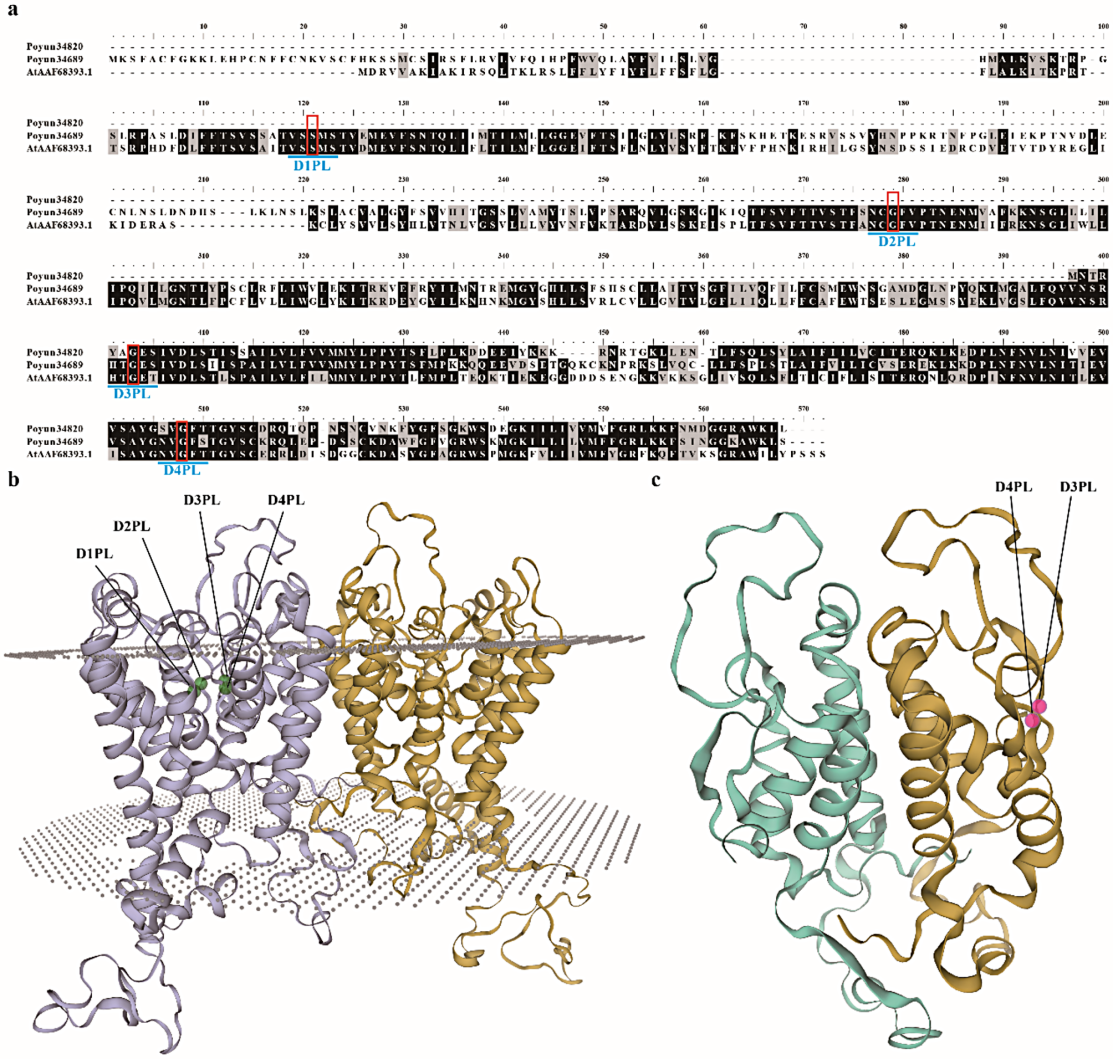
Sequence and Structure analysis of *P. yunnanenesis* HKTs. (**a**) Sequence alignment of PyHKTs and AtHKT1. DP1L, DP2L, DP3L, and DP4L were 4 P-loops of HKTs, which marked with bright blue lines, characteristic Glycine and Serine were marked with red box. The black shading revealed same amino acid, the grey shading revealed the similarity threshold over 60%. (**b**) 3D Structure of Poyun34689. The Glycine and Serine sites were labled with green balls. (**c**) 3D Structure of Poyun34820. The Glycine and Serine sites were labled with light pink balls. The 3D reconstruction map of PyHKTs were built with SWISS-MODEL Workspace of Expasy [27]

To further investigate poplar HKTs, we conducted sequence alignment and structural analysis of 16 poplar HKTs. All poplar HKTs were found to contain four conserved pore loop transmembrane domain motifs featuring hallmark selectivity filter residues (SGGG), characteristic of Class I HKTs (Figure S1) [10]. On the basis of phylogenetic analysis and sequence similarity, the 16 poplar HKTs can be classified into two groups (Fig. 1, Figure S2). Two HKTs from *P. yunnanensis* were divided into two different groups, which were representative for structural analysis (Figure S3). Protein models of Poyun34689 were constructed on the basis of 8w9n.1. The sodium transporter HKT1 template (structure of AtHKT1;1 in NaCl at 2.7 Å resolution) exhibited 52.63% sequence identity, a GMQE (global model quality estimate) of 0.66, and a QMEANDisCo global score of 0.71 ± 0.05 (Fig. 1b). Conversely, protein models of Poyun34820 were constructed via 8w9n.1. A sodium transporter HKT1 template (structure of AtHKT1;1 in NaCl at 2.7 Å resolution) exhibited 56.36% sequence identity, a GMQE of 0.64, and a QMEANDisCo global score of 0.61 ± 0.05 (Fig. 1c). The length of the Poyun34820 protein was significantly shorter than that of the Poyun34689 protein, which lacked two characteristic transmembrane domain motifs (D1PL and D2PL) (Table 1; Fig. 1c). The same structural template of the two groups of PyHKTs revealed structural similarity, which was also conserved across all poplars (Figure S3). Six poplar HKTs (XP_011017751, KAG6740023, XP_034918867, KAG6741040, Podel.18G140400, Potri.018G132200) exhibited structural similarity to the protein model of Poyun34689. Conversely, XP_011017602.1, XP_011017603.1, XP_011017604.1, XP_011017605.1, XP_034906209.1, Podel.18G152700.1, KAG6741120.1, Potri.018G147501.2 exhibited structural similarity to the protein model of Poyun34820. On the basis of this analysis, two groups of HKTs were identified among the 16 poplar HKTs. Although, HKT protein numbers were various among different poplar species, 5 *P. euphratica* HKTs also classified to two groups according to their sequence similarity (Figure S4).

.

### Characteristic protein and gene structure of poplar HKTs

**Fig. 2:**
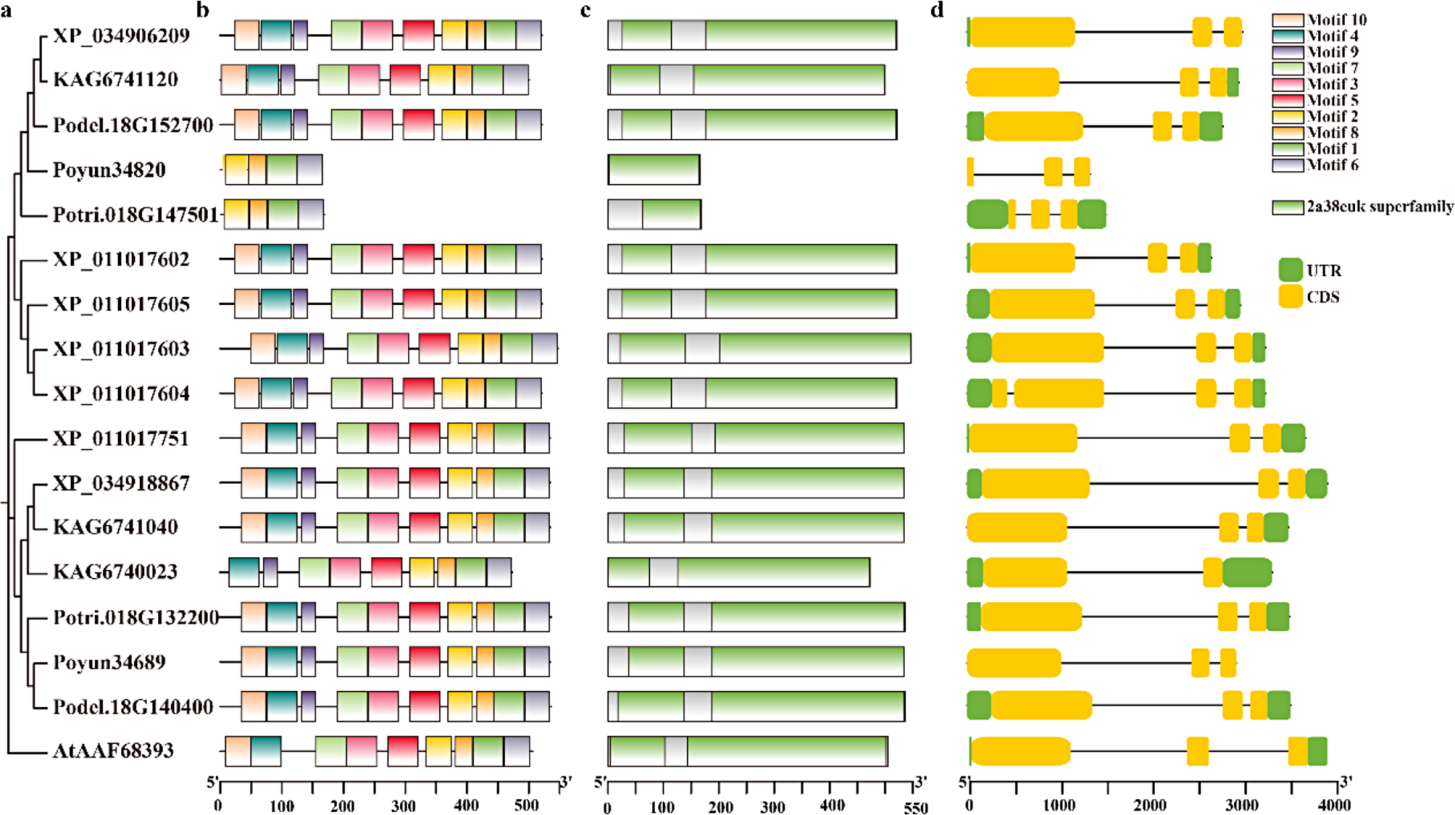
Protein and gene structures of poplar HKT proteins. (**a**) Physiology tree of poplar HKT proteins and AtHKT1. The physiology tree was constructed using MEGA11 (version 11.0.9) with the Maximum Likelihood method. (**b**) Conserved motifs identified in poplar HKT proteins and AtHKT1. Motifs were identified using MEME, utilizing a threshold of ten motifs. Ten motif labels are listed on the right. (**c**) Conserved domains in poplar HKT proteins and AtHKT1. Green boxes represent the 2a38euk superfamily. (**d**) Gene structures of poplar HKTs and AtHKT1. Green box represents UTR, while yellow box represents CDSs

To further investigate the structure and sequence characteristics, we analyzed the conserved motifs, domains and gene structures of all poplar HKTs and AtHKT (Fig. 2). Poyun34820 and Potri.018G147501 were shorter than the other identified HKTs and contained only four conserved motifs in the 3’ regions of other poplar HKTs (motif2, motif8, motif3, and motif9) (Fig. 2b). All nine identified motifs of *Arabidopsis* were also conserved in most poplar HKTs, except for KAG6740023, which lacked motif 10 of the 5’ end in the majority of HKTs. Conversely, most poplar HKTs contained a unique conserved motif (motif 9), which was not identified in AtHKT. The four conserved transmembrane loop motifs were also identified in the conserved motifs of poplar, specifically G-S-S-S, which were located in motif 4 (S), motif 3 (G), motif 2 (G), and motif 1 (G), respectively. Poyun34820 and Potri.018G147501 contained only one conserved 2a38euk, which was fewer than those in other poplar HKTs. With the exceptions of Poyun34820 and Potri.018G147501, *Arabidopsis* HKT1 and other poplar HKTs all contained two conserved 2a38euk domains, which were separated by several amino acids (Fig. 2c). With the exception of the shorter HKTs (Poyun34820 and Potri.018G147501), most HKTs feature a long exon and several short exons in their gene structures. The UTR regions, as identified by the annotation message, exhibited differences among the various HKTs, with variability in their lengths and positions (Fig. 2d).

**Fig. 3:**
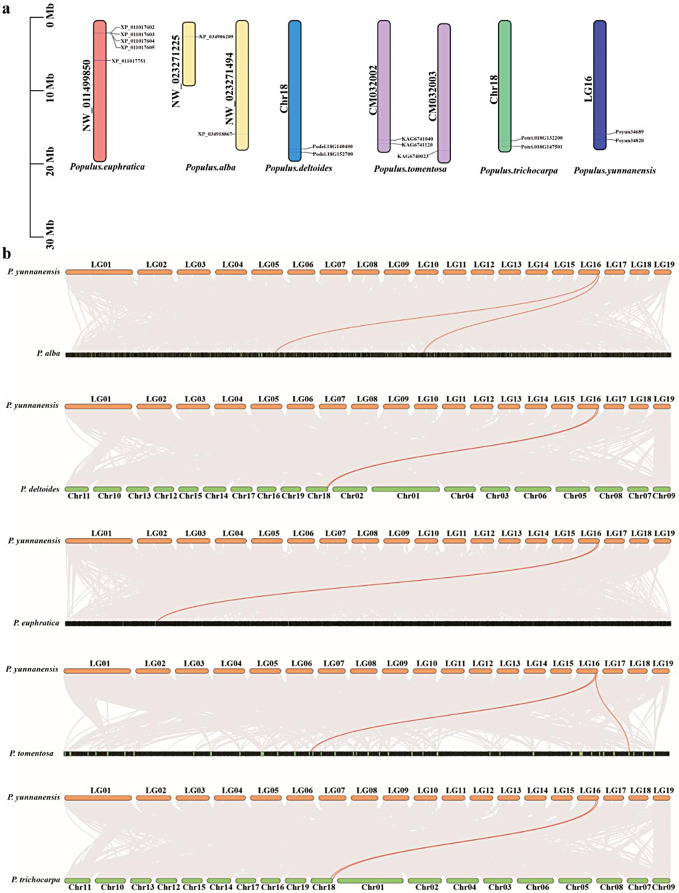
Chromosome location and collinearity analysis of poplar HKTs. (**a**) Chromosome location of 16 poplar *HKT*s. (**b**) Collinearity analysis of poplar HKTs with *PyHKT*s. Red lines reveal the Collinearity pairs between poplar *HKT*s

The distribution of HKTs varied among the different poplars. Five HKTs of *P. euphratica* and two HKTs each of *P. deltoides*, *P. trichocarpa*, and *P. yunnanensis* were distributed on one chromosome, indicating their conserved similarity and distribution. Three HKTs of *P. tomentosa* and two HKTs of *P. alba* were distributed on two chromosomes, revealing the differentiation of their HKTs (Fig. 3a). In the collinearity analysis, all poplar HKTs had collinearity relationship with two *PyHKT*s, which revealed that tandem duplication events occurred between poplar HKTs (Fig. 3b) [29]. In the evolutionary analysis, the Ka/Ks values for different poplar HKTs were all less than one, indicating that HKT genes underwent negative selection to eliminate deleterious mutations and preserve the original protein function (Table S2) [30].

### Cis-elements of poplar HKTs on their promoters and their expression

**Fig. 4:**
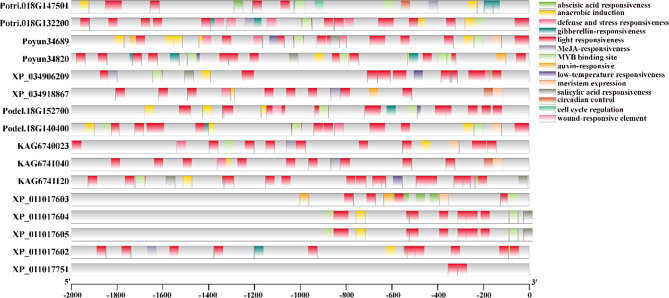
Cis-elements within the promoter regions of poplar HKT genes. Different color boxes represent cis-elements identified in the upstream 2000 bp sequences of the transcription start sites of poplar HKT genes

To predict the function of poplar HKTs, the cis-elements in their promoters were detected (Fig. 4). Light-responsive elements were the most common cis-elements in the promoters of poplar HKTs and were detected in all the members. Abscisic acid (ABA) is an important regulatory hormone in plant growth and stress response [31]. ABA-responsive elements were detected in all poplar HKTs, but differences in the number of cis-elements between species were detected [32]. Many ABA-responsive elements were detected in all *P. tomentosa* and *P. trichocarpa* HKT promoters, but several were detected in the HKTs of other poplars. Anaerobic induction elements were commonly detected in all poplar HKTs, except for two *P. euphratica* HKT members (XP_011017603, XP_011047751). Many stress-responsive elements, which vary among different species, have been detected in poplar HKTs. Salicylic acid (SA)-responsive elements were detected in the HKTs of five *Populus* species, with the exception of *P. trichocarpa.* MeJA-responsive elements were detected in six poplar HKTs across four *Populus* species. Low-temperature-responsive elements were also detected in four *Populus* species. Cis-elements related to plant growth regulation were detected in some poplar HKTs, including auxin-responsive elements, cell cycle regulation elements, circadian control elements, gibberellin (GA)-responsive elements, and meristem expression elements. MYB binding sites, which are important transcription factors in plant growth and stress response, were detected in the HKTs of all *Populus* species. These results revealed the differentiation of HKTs among poplars [33].

**Fig. 5:**
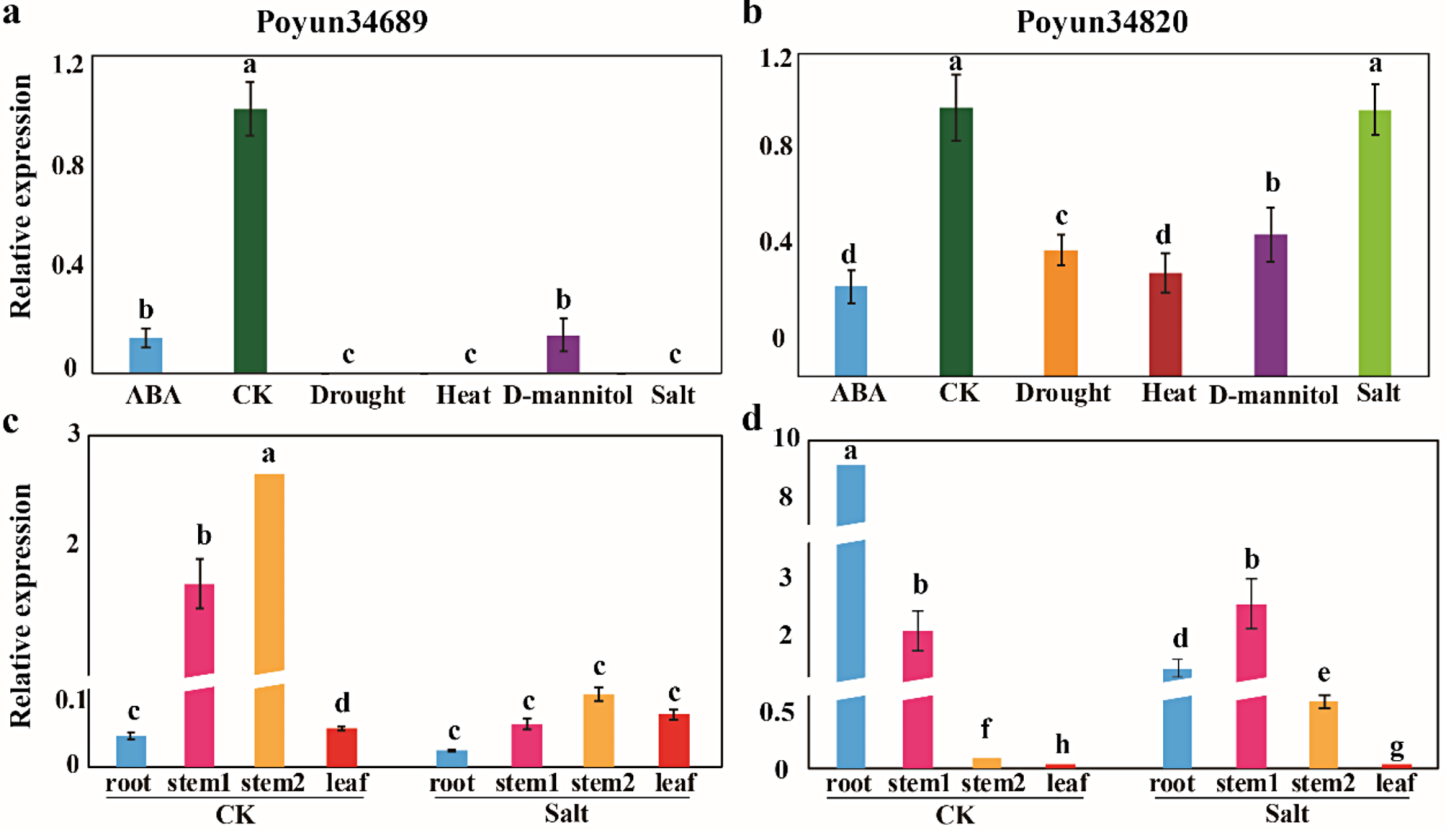
Relative expression of *PyHKTs* in different tissues under various stress conditions. **a** and **b**. Relative expression of *Poyun34689* and *Poyun34820* under various stress conditions. Blue box represents ABA treatment; dark green box indicates the untreated control; orange box denotes drought treatment; red box represents heat treatment; violet box corresponds to D-mannitol treatment; olive green box denotes salt treatment. c and d. Relative expression of *Poyun34689* and *Poyun34820* in various tissues. Blue box represents root tissue; rose red box indicates stem1 (xylem); orange box denotes stem2 (phloem); red box represents leaf tissue. The left group displays expression level under normal conditions, while the right group illustrates expression levels under salt stress

The expression of HKTs in *P. trichocarpa* was not detected in different tissues or under hormone treatments, except in the roots (Figure S5). To predict the function of HKTs in *P. yunnanensis*, the expression of two *PyHKT*s was measured via qRT-PCR (Fig. 5). *Poyun34689* was expressed at lower levels in all the tested *P. yunnanensis* tissues, with an even lower expression trend than that in the untreated samples (Fig. 5a and b). The expression of *Poyun34820* was slightly greater under the different stress treatments, especially under salt stress, but was also slightly lower or not significantly different from that in the untreated control conditions (Fig. 5c and d). To predict the function of *PyHKT*s under salt stress, their expression was tested in different tissues under salt stress. *Poyun34689* presented relatively high expression in stem1 (xylem) and stem2 (phloem) under normal conditions, which decreased under salt stress (Fig. 5c). *Poyun34820* was more highly expressed in roots and xylem under both untreated and salt stress conditions, while its expression in phloem under salt stress was also high (Fig. 5d). The above results revealed that the expression of *PyHKT*s was tissue-specific and influenced by salt stress.

### Proteins predicted to interact with poplar HKTs

**Fig. 6:**
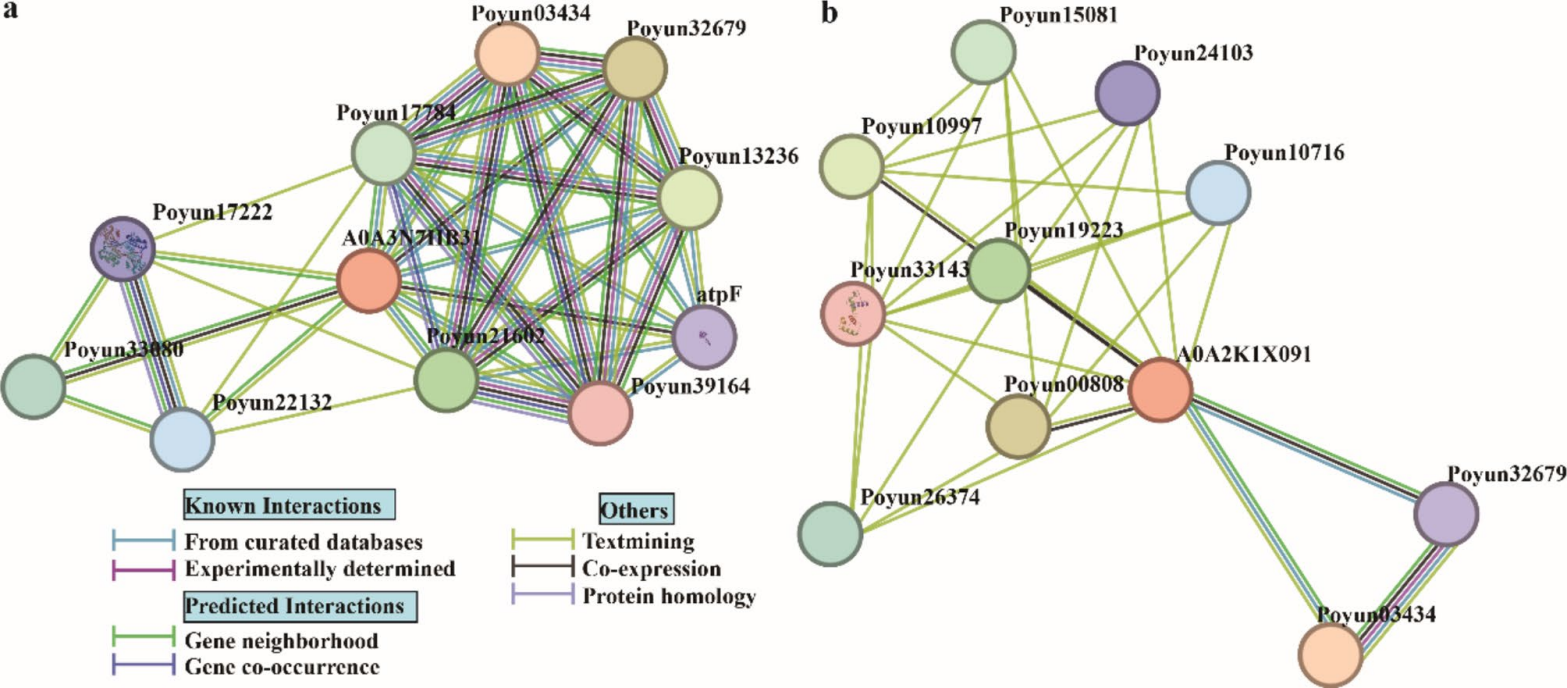
Predicted protein interaction network of poplar HKTs. The various nodes represent distinct proteins. The core proteins A0A3N7HB31 (**a**) and A0A2K1 × 091 (**b**) represent the most similar HKT models of poplar as predicted by STRING. The lines of varying colors illustrate different modes of interaction. The predicted modes of interaction are detailed in the lower left corner

To further explore the function and interaction network of poplar HKTs, we predicted the interaction proteins of poplar HKTs using STRING [34]. All 16 poplar HKTs were found to be similar to two HKTs of poplar (A0A3N7HB31 and A0A2K1 × 091). In fact, one of the two detected HKTs from *P. trichocarpa* and *P. yunnanensis* (Potri.018G147501, Poyun34820) was classified similarly to A0A3N7HB31 (Fig. 6a). All other poplar HKTs were more similar to the other poplar HKT model (A0A2K1 × 091) (Fig. 6b). During the prediction of interacting proteins, ATPase (Poyun32679, Poyun03434) was commonly found in both poplar HKT models (Table S3). Transporter proteins, such as the aluminum-activated malate transporter in A0A2K1 × 091 (Poyun10997, Poyun19223, Poyun00808) and the cation transport protein in A0A3N7HB31 (Poyun34820), were also detected multiple times. A proline biosynthesis protein (P5CS, Poyun17222, Poyun22132) was detected to interact with A0A3N7HB31. MYB DNA-binding protein (Poyun33143) was found to interact with A0A2K1 × 091. The expression of genes encoding predicted functional proteins in *P. trichocarpa* was also induced by hormone and stress treatment (Figure S6).

## Discussion

HKT channels, which are plant-specific proteins, play crucial roles in plant Na^+^ and K^+^ uptake, as well as in maintaining Na^+^-K^+^ homeostasis [5]. With the development of sequencing technology, members of HKT families have been identified in many different plants, such as *Mocho de Espiga Branca*, *Sorghum bicolor*, *Oryza sativa*, *Triticum monoccocum* and *Thellungiella salsuginea*. These HKTs play functional roles in plant growth and stress response [11–13, 35]. However, little is known about the comparative analysis, structural characteristics and functional differentiation of HKTs in *Populus* species, which are widely distributed forest trees around the world [20]. In this study, a total of 16 HKTs were identified in six *Populus* species, all of which belong to Class I HKTs on the basis of their structure and physiological relationship with *Arabidopsis* HKT1 [9]. The variation in copy numbers and chromosomal locations of *Populus HKT*s revealed genome duplication events and functional differentiation. The expression profile indicated functional differentiation of poplar HKTs across different tissues and under various stresses, especially for two PyHKTs, which may function with their predicted cation transporters and enzyme proteins.

Recently, 3 HKTs, 6 HKTs, 2 HKTs, 5 HKTs, 2 HKTs, and 4 HKTs were detected in *Cucumis sativus* L., *Vitis vinifera*, *Zea mays*, *Glycine max*, *Solanum lycopersicum*, and *Sorghum bicolor*, which revealed the various HKTs present in these herbaceous plants [36]. There is insufficient research on the distribution of HKTs in xylophyta. To investigate the distribution and function of HKTs in forest trees, 16 HKTs were detected in six *Populus* species. Poplar is a model forest tree distributed across the world that exhibits phenotypic and adaptive divergence [20]. The six *Populus* species analyzed for HKTs presented diverse genomic physiological relationships and sizes, which may account for the variation in HKT numbers among different *Populus* species [20]. All the evolutionarily related *Populus* species (Clade-I: *P. yunnanensis*, *P. trichocarpa*, *P. deltoides*) presented two HKTs; *P. alba*, which represented Clad-II, also presented two HKTs; however, the Clade-III *Populus* species (*P. euphratica*) presented five HKTs, which was more than those of the other species (Table 1) [20]. The similar number of HKTs, especially among evolutionarily related *Populus* species, suggested that HKT proteins were conserved within the *Populus* genus. Unlike the five subfamilies of HKTs classified in herbaceous plants, all 16 *Populus* HKTs belong to the same clade as AtHKT, representing Class I HKTs (Figure S1) [9]. The Ka/Ks ratios of all *HKT*s from *Populus* species that we calculated were all less than one, indicating that these genes have undergone strong purifying selective pressure, suggesting their stable function through evolution. This is consistent with the gradual evolutionary trend of HKTs and the retention of HKT class I in dicotyledons (Table S2) [5, 30]. In addition to the conservation of HKTs within the *Populus* genus, some HKTs presented species-specific characteristics, such as Potri.018G147501 (*P. trichocarpa*) and Poyun34820 (*P. yunnanensis*), which presented similar low protein weights, specific subcellular locations, and higher Ka/Ks ratios (0.61) than other poplar HKTs did (Table 1, Table S2). Furthermore, 16 HKTs were classified into two groups on the basis of their phylogenetic relationships, similar to the classification of Class I HKTs among different species (Figure S2) [37]. However, their sequences were conserved and contain four pore loops, including the class I HKT characteristic SGGG motif, which is a functional feature of the transmembrane structure of ion channel proteins (Fig. 1, Figure S3) [5]. The crystal structure of *Populus* HKTs was constructed on the basis of the same *Arabidopsis* HKT models (Fig. 1), which exhibit conserved transmembrane loops and indicate conservation across species [38]. Moreover, Potri.018G147501 and Poyun34820 were exceptions, each containing only two conserved loops located at the C-terminus (Fig. 1, Figure S3) [39]. During plant evolution, short HKTs have been widely distributed among several species, playing a fundamental role in stress responses. The reasons behind this distribution, however, remain understudied [36]. All 16 poplar HKTs were within two types of protein HKT structures, which underscores the conservation of poplar HKTs. Specifically, four HKT genes from *P. euphratica* exhibited highly similar protein sequences compared to the fifth *P. euphratica* HKT (Figure S4). The structure and sequence differentiation may be related to gene evolution, given that their Ka/Ks ratios were greater than 0.5 and higher than those of other poplar HKTs (Figure S3, Table S2) [40].

Except for Potri.018G147501 and Poyun34820, all the remaining 14 conserved poplar HKTs contained 8 motifs, including four transmembrane loops (Fig. 2) [5]. Potri.018G147501 and Poyun34820 contained four conserved motifs at the C-terminus, attributed to their shorter sequences (Table 1; Fig. 1). Conversely, the detected conserved motifs in poplar HKTs differed from those in Arabidopsis, with the latter lacking motif 9 located between two transmembrane domains (Fig. 1) [5]. Similarly, *Populus* HKTs also contained the same conserved two-domain structure (pore-loop transporter domain) as *Arabidopsis* HKTs did, with the exception of Potri.018G147501 and Poyun34820 (Fig. 1). The number of exons varied among the poplar *HKT*s. The three-exon structure (a long exon and two short exons) was the most common gene structure among *Populus HKT*s and was detected in 13 poplar *HKT*s, including all members of group II. The HKT members of group I presented various gene structures, notably, *XP_011017604* (*P. euphratica*), *Potri.018G147501* and *Poyun34820* (Fig. 2). During evolution, genome duplication was the primary reason for changes in genome size and gene family duplication [41]. *Populus* species have experienced genome duplication, chromosomal rearrangement, gene duplication and adaptive evolution [20, 42]. Most of the 16 poplar HKTs appeared on the same chromosome or assembly scaffold, which was not associated with gene number. These results aligned with the distribution of HKTs among nine different species (Fig. 3a) [36]. The collinearity analysis was consistent with the two groups classified based on protein 3D structure, during which all 14 poplar HKTs in the other five poplars exhibited collinear pairs with two *P. yunnanensis* HKTs (Figure S3b). Tandem duplication may be the primary reason for the gene family formation of poplar HKTs [29]. On the other hand, the genome assembly level also influenced the identification of gene copy number. The increased HKT numbers in *P. euphratica* may be attributed to the scaffold assembly level of the genome [43].

Gene expression is always regulated by spatiotemporal signals, such as the environment and regulators inside plants [44]. The promoter regions of target genes are key regulatory DNA elements that can be regulated by transcriptional enhancers [45]. The identification of cis-elements in gene promoters is an effective way to predict regulators of gene function and expression [46]. Among the promoter regions of poplar HKTs, light-responsive elements were the most widely distributed elements. Additionally, hormone (ABA, SA, Me-JA, and auxin)-responsive, stress (anaerobic and low-temperature)-responsive, and stress-related transcription factor (MYB) genes were detected (Fig. 4). The detected cis-elements may imply that the functions of poplar HKTs are related to plant regulation and the stress response. In *Sorghum bicolor*, SbHKT1;4 can maintain the optimal Na^+^/K^+^ balance under salt (Na^+^) stress, which is upregulated in salt-tolerant species [12]. Poplar *PeHKT1;1* is expressed mainly in stems and roots and is strongly induced by salt stress. Overexpression of this gene enhances plant salt tolerance through increased antioxidant capacity [13]. In our research, the expression of *P. yunannensis HKT* (*Poyun34820*) was induced by salt stress compared with other stress treatments and was not significantly different from that of the untreated control in the leaves. Compared with that in the untreated control leaves, the expression of the other *P. yunannensis HKT* (*Poyun34689*) in the leaves was significantly inhibited by stress treatment (Fig. 5). Furthermore, the expression levels of *P. yunannensis HKT*s were greater in the roots and stems (xylem) than in the leaves, particularly *Poyun34820*, which also increased under salt stress, which was consistent with the expression pattern of *P. trichocarpa* HKTs (Figure S5). Similar results were also reported in *Spartina alterniflora*, where *SaHKT*s were differentially expressed in specific tissues under salt stress [37]. The expression of the two *P. yunannensis HKT*s was different, similar to the differentiation of HKTs in grape and soybean [36, 47]. This suggests that neofunctionalization may have occurred during the evolution of poplar HKTs.

To explore the function of HKTs, the prediction of interacting functional proteins may provide valuable insights [48]. A comparison of two different nearest protein models (A0A3N7HB31 and A0A2K1 × 091) with poplar HKTs revealed different structural variations and interactions (Fig. 6) [34]. H^+^-ATPase is an important ATP-driven proton pump that generates a proton motive force for secondary active transport, which is crucial for plant growth and stress adaptation [49]. The interaction between ATPase and poplar HKTs elucidated the functionality of HKTs under salt stress by enhancing the proton motive force with increased expression of ATPase (Table S3, Figure S6). Ion transporters serve as crucial regulators of plant stress responses by modulating ion flow during abiotic stress [50, 51]. The predicted transporters interacting with poplar HKTs also confirmed the functionality of HKTs by regulating ion transport activity during plant growth and stress adaptation (Figure S6).

## Conclusion

HKTs play important roles in plant ion transport regulation and stress response. In this study, we identified 16 HKTs in six *Populus* species across various evolutionary clades. All poplar HKTs were classified as Class I HKTs on the basis of their physiological relationships and conserved amino acids in the loop structures, which aligned with gene evolutionary conservation. On the basis of their physiological relationships, conserved motifs, domains and gene structures, poplar HKTs can be classified into two subgroups, similar to the differentiation of HKT homologs in the same *Populus* species. The difference in the number of HKT homologs and chromosomal locations contributed to the differentiation of poplar HKTs during their evolution. Functional prediction and expression analyses revealed that HKT exhibited increased expression in root and stem tissues under salt stress, with variability observed among different homologs, which may interact with their predicted regulators and partners. Our research provides a novel perspective on the evolutionary and functional differentiation of HKTs in six *Populus* species. However, the specific regulatory mechanisms of HKTs in various tissues and under stress conditions require further investigation in future studies.

## Electronic supplementary material

Below is the link to the electronic supplementary material.

Supplementary Material 1: Physiology tree of 16 *Populus* HKT proteins, AtHKT1, and TaHKT2;1

Supplementary Material 2: Sequence alignment of 16 HKTs from six *Populus* species

Supplementary Material 3: 3D structure analysis of two subgroup HKTs of six *Populus* species

Supplementary Material 4: Sequence alignment of 5 HKTs from *P. euphratica*

Supplementary Material 5: Heatmap of two HKTs from *P. trichocarpa* under different tissues and treatments

Supplementary Material 6: Heatmap of predicted interaction protein homologs in *P. trichocarpa* during different tissues and treatments

Supplementary Material 7: qRT-PCR primers for the *P. yunnanensis* HKT genes

Supplementary Material 8: Ka/Ks ratios of different poplar *HKT* genes

Supplementary Material 9: Information on the proteins predicted to interact with *P. yunnanensis* HKTs

## Data Availability

The datasets generated and analyzed during the current study, including the HKT sequences and annotations, are publicly available in the DNA Data Bank of Japan (DDBJ, https://www.ddbj.nig.ac.jp/index-e.html) and the NCBI GenBank (National Center for Biotechnology Information, https://www.ncbi.nlm.nih.gov/nuccore/),wit),with the accession numbers LC848849-LC848864.

## Abbreviations

HKT High: Affinity K^+^ transporter
ABA: Abscisic acid
RNA: Ribonucleic acid
MW: Molecular weights
pI: Isoelectric points
GRAVY: Grand average of hydropathicity
qRT-PCR: Quantitative reverse transcription polymerase chain reaction
SA: Salicylic acid
GA: Gibberellin

## References

[1] Hauser F, Horie T. A conserved primary salt tolerance mechanism mediated by HKT transporters: a mechanism for sodium exclusion and maintenance of high K(^+^)/Na(^+^) ratio in leaves during salinity stress. Plant Cell Environ. 2010;33(4):552–65. 19895406 10.1111/j.1365-3040.2009.02056.x

[2] Deinlein U, Stephan AB, Horie T, Luo W, Xu G, Schroeder JI. Plant salt-tolerance mechanisms. Trends Plant Sci. 2014;19(6):371–9. 24630845 10.1016/j.tplants.2014.02.001 PMC4041829

[3] Sahi C, Singh A, Blumwald E, Grover A. Beyond osmolytes and transporters: novel plant salt-stress tolerance-related genes from transcriptional profiling data. Physiol Plant. 2006;127(1):1–9.

[4] Kato N, Akai M, Zulkifli L, Matsuda N, Kato Y, Goshima S, Hazama A, Yamagami M, Guy HR, Uozumi N. Role of positively charged amino acids in the M2D transmembrane helix of Ktr/Trk/HKT type cation transporters. Channels (Austin Tex). 2007;1(3):161–71. 18690031 10.4161/chan.4374

[5] Riedelsberger J, Miller JK, Valdebenito-Maturana B, Piñeros MA, González W, Dreyer I. Plant HKT channels: an updated view on structure, function and Gene Regulation. Int J Mol Sci 2021, 22(4). 10.3390/ijms22041892 PMC7918770 33672907

[6] Mäser P, Hosoo Y, Goshima S, Horie T, Eckelman B, Yamada K, Yoshida K, Bakker EP, Shinmyo A, Oiki S, et al. Glycine residues in potassium channel-like selectivity filters determine potassium selectivity in four-loop-per-subunit HKT transporters from plants. Proc Natl Acad Sci USA. 2002;99(9):6428–33. 11959905 10.1073/pnas.082123799 PMC122965

[7] Horie T, Brodsky DE, Costa A, Kaneko T, Lo Schiavo F, Katsuhara M, Schroeder JI. K^+^ transport by the OsHKT2;4 transporter from rice with atypical Na^+^ transport properties and competition in permeation of K^+^ over Mg^2+^ and Ca^2+^ ions. Plant Physiol. 2011;156(3):1493–507. 21610181 10.1104/pp.110.168047 PMC3135959

[8] Horie T, Hauser F, Schroeder JI. HKT transporter-mediated salinity resistance mechanisms in Arabidopsis and monocot crop plants. Trends Plant Sci. 2009;14(12):660–8. 19783197 10.1016/j.tplants.2009.08.009 PMC2787891

[9] Uozumi N, Kim EJ, Rubio F, Yamaguchi T, Muto S, Tsuboi A, Bakker EP, Nakamura T, Schroeder JI. The *Arabidopsis* HKT1 gene homolog mediates inward na(^+^) currents in xenopus laevis oocytes and na(^+^) uptake in Saccharomyces cerevisiae. Plant Physiol. 2000;122(4):1249–59. 10759522 10.1104/pp.122.4.1249 PMC58961

[10] Wang J, Luo Y, Ye F, Ding ZJ, Zheng SJ, Qiao S, Wang Y, Guo J, Yang W, Su N. Structures and ion transport mechanisms of plant high-affinity potassium transporters. Mol Plant. 2024;17(3):409–22. 38335958 10.1016/j.molp.2024.01.007

[11] Borjigin C, Schilling RK, Bose J, Hrmova M, Qiu J, Wege S, Situmorang A, Byrt C, Brien C, Berger B, et al. A single nucleotide substitution in TaHKT1;5-D controls shoot na(+) accumulation in bread wheat. Plant Cell Environ. 2020;43(9):2158–71. 32652543 10.1111/pce.13841 PMC7540593

[12] Wang TT, Ren ZJ, Liu ZQ, Feng X, Guo RQ, Li BG, Li LG, Jing HC. SbHKT1;4, a member of the high-affinity potassium transporter gene family from Sorghum bicolor, functions to maintain optimal Na⁺ /K⁺ balance under Na⁺ stress. J Integr Plant Biol. 2014;56(3):315–32. 24325391 10.1111/jipb.12144

[13] Xu M, Chen C, Cai H, Wu L. Overexpression of PeHKT1;1 improves Salt Tolerance in Populus. Genes 2018, 9(10). 10.3390/genes9100475 PMC6210203 30274294

[14] Byrt CS, Xu B, Krishnan M, Lightfoot DJ, Athman A, Jacobs AK, Watson-Haigh NS, Plett D, Munns R, Tester M, et al. The na(+) transporter, TaHKT1;5-D, limits shoot na(^+^) accumulation in bread wheat. Plant Journal: Cell Mol Biology. 2014;80(3):516–26. 10.1111/tpj.12651 25158883

[15] Ali Z, Park HC, Ali A, Oh DH, Aman R, Kropornicka A, Hong H, Choi W, Chung WS, Kim WY, et al. TsHKT1;2, a HKT1 homolog from the extremophile *Arabidopsis* relative Thellungiella salsuginea, shows K(^+^) specificity in the presence of NaCl. Plant Physiol. 2012;158(3):1463–74. 22238420 10.1104/pp.111.193110 PMC3291249

[16] Horie T, Sugawara M, Okada T, Taira K, Kaothien-Nakayama P, Katsuhara M, Shinmyo A, Nakayama H. Rice sodium-insensitive potassium transporter, OsHAK5, confers increased salt tolerance in tobacco BY2 cells. J Biosci Bioeng. 2011;111(3):346–56. 21084222 10.1016/j.jbiosc.2010.10.014

[17] Tounsi S, Ben Amar S, Masmoudi K, Sentenac H, Brini F, Véry AA. Characterization of two HKT1;4 transporters from Triticum monococcum to elucidate the determinants of the Wheat Salt Tolerance Nax1 QTL. Plant Cell Physiol. 2016;57(10):2047–57. 27440547 10.1093/pcp/pcw123

[18] Tounsi S, Saïdi MN, Abdelhedi R, Feki K, Bahloul N, Alcon C, Masmoudi K, Brini F. Functional analysis of TmHKT1;4-A2 promoter through deletion analysis provides new insight into the regulatory mechanism underlying abiotic stress adaptation. Planta. 2021;253(1):18. 33392811 10.1007/s00425-020-03533-9

[19] Xu B, Hrmova M, Gilliham M. High affinity na(^+^) transport by wheat HKT1;5 is blocked by K(). Plant Direct. 2020;4(10):e00275. 33103046 10.1002/pld3.275 PMC7576878

[20] Shi T, Zhang X, Hou Y, Jia C, Dan X, Zhang Y, Jiang Y, Lai Q, Feng J, Feng J, et al. The super-pangenome of *Populus* unveils genomic facets for its adaptation and diversification in widespread forest trees. Mol Plant. 2024;17(5):725–46. 38486452 10.1016/j.molp.2024.03.009

[21] Jansson S, Douglas CJ. *Populus*: a model system for plant biology. Annu Rev Plant Biol. 2007;58:435–58. 17280524 10.1146/annurev.arplant.58.032806.103956

[22] Ahkami AH. Systems biology of root development in *Populus*: review and perspectives. Plant Science: Int J Experimental Plant Biology. 2023;335:111818. 10.1016/j.plantsci.2023.111818 37567482

[23] Porth I, El-Kassaby YA. Using *Populus* as a lignocellulosic feedstock for bioethanol. Biotechnol J. 2015;10(4):510–24. 25676392 10.1002/biot.201400194

[24] Tamura K, Stecher G, Kumar S. MEGA11: Molecular Evolutionary Genetics Analysis Version 11. Mol Biol Evol. 2021;38(7):3022–7. 33892491 10.1093/molbev/msab120 PMC8233496

[25] Letunic I, Khedkar S, Bork P. SMART: recent updates, new developments and status in 2020. Nucleic Acids Res. 2021;49(D1):D458–60. 33104802 10.1093/nar/gkaa937 PMC7778883

[26] Wang J, Chitsaz F, Derbyshire MK, Gonzales NR, Gwadz M, Lu S, Marchler GH, Song JS, Thanki N, Yamashita RA, et al. The conserved domain database in 2023. Nucleic Acids Res. 2023;51(D1):D384–8. 36477806 10.1093/nar/gkac1096 PMC9825596

[27] Waterhouse A, Bertoni M, Bienert S, Studer G, Tauriello G, Gumienny R, Heer FT, de Beer TAP, Rempfer C, Bordoli L, et al. SWISS-MODEL: homology modelling of protein structures and complexes. Nucleic Acids Res. 2018;46(W1):W296–303. 29788355 10.1093/nar/gky427 PMC6030848

[28] Li P, Wang J, Jiang D, Yu A, Sun R, Liu A. Function and Characteristic Analysis of Candidate PEAR Proteins in *Populus yunnanensis*. Int J Mol Sci 2023, 24(17). 10.3390/ijms241713101 PMC10488302 37685908

[29] Wu F, Zhao M, Zhang Y, Si W, Cheng B, Li X. Systematic analysis of the *Rboh* gene family in seven gramineous plants and its roles in response to arbuscular mycorrhizal fungi in maize. BMC Plant Biol. 2023;23(1):603. 38030972 10.1186/s12870-023-04571-7 PMC10688149

[30] Liao B, Wang C, Li X, Man Y, Ruan H, Zhao Y. Genome-wide analysis of the *Populus trichocarpa* laccase gene family and functional identification of PtrLAC23. Front Plant Sci. 2022;13:1063813. 36733583 10.3389/fpls.2022.1063813 PMC9887407

[31] Kuromori T, Seo M, Shinozaki K. ABA Transport and Plant Water stress responses. Trends Plant Sci. 2018;23(6):513–22. 29731225 10.1016/j.tplants.2018.04.001

[32] You Z, Guo S, Li Q, Fang Y, Huang P, Ju C, Wang C. The CBL1/9-CIPK1 calcium sensor negatively regulates drought stress by phosphorylating the PYLs ABA receptor. Nat Commun. 2023;14(1):5886. 37735173 10.1038/s41467-023-41657-0 PMC10514306

[33] Yang L, Chen Y, Xu L, Wang J, Qi H, Guo J, Zhang L, Shen J, Wang H, Zhang F, et al. The OsFTIP6-OsHB22-OsMYBR57 module regulates drought response in rice. Mol Plant. 2022;15(7):1227–42. 35684964 10.1016/j.molp.2022.06.003

[34] Szklarczyk D, Kirsch R, Koutrouli M, Nastou K, Mehryary F, Hachilif R, Gable AL, Fang T, Doncheva NT, Pyysalo S, et al. The STRING database in 2023: protein-protein association networks and functional enrichment analyses for any sequenced genome of interest. Nucleic Acids Res. 2023;51(D1):D638–46. 36370105 10.1093/nar/gkac1000 PMC9825434

[35] Wang X, Shen X, Qu Y, Zhang H, Wang C, Yang F, Shen H. Structural insights into ion selectivity and transport mechanisms of *Oryza sativa* HKT2;1 and HKT2;2/1 transporters. Nat Plants. 2024;10(4):633–44. 38570642 10.1038/s41477-024-01665-4

[36] Li H, Xu G, Yang C, Yang L, Liang Z. Genome-wide identification and expression analysis of HKT transcription factor under salt stress in nine plant species. Ecotoxicol Environ Saf. 2019;171:435–42. 30639869 10.1016/j.ecoenv.2019.01.008

[37] Yang M, Chen S, Huang Z, Gao S, Yu T, Du T, Zhang H, Li X, Liu CM, Chen S, et al. Deep learning-enabled discovery and characterization of HKT genes in *Spartina alterniflora*. Plant Journal: Cell Mol Biology. 2023;116(3):690–705. 10.1111/tpj.16397 37494542

[38] Hamamoto S, Horie T, Hauser F, Deinlein U, Schroeder JI, Uozumi N. HKT transporters mediate salt stress resistance in plants: from structure and function to the field. Curr Opin Biotechnol. 2015;32:113–20. 25528276 10.1016/j.copbio.2014.11.025

[39] Haro R, Bañuelos MA, Rodríguez-Navarro A. High-affinity sodium uptake in land plants. Plant Cell Physiol. 2010;51(1):68–79. 19939835 10.1093/pcp/pcp168

[40] Khan N, You FM, Datla R, Ravichandran S, Jia B, Cloutier S. Genome-wide identification of ATP binding cassette (ABC) transporter and heavy metal associated (HMA) gene families in flax (*Linum usitatissimum* L). BMC Genomics. 2020;21(1):722. 33076828 10.1186/s12864-020-07121-9 PMC7574471

[41] Jiao Y. Double the genome, double the Fun: genome duplications in Angiosperms. Mol Plant. 2018;11(3):357–8. 29476919 10.1016/j.molp.2018.02.009

[42] Tuskan GA, Difazio S, Jansson S, Bohlmann J, Grigoriev I, Hellsten U, Putnam N, Ralph S, Rombauts S, Salamov A et al. The genome of black cottonwood, *Populus trichocarpa* (Torr. & Gray). *Science (New York, NY)* 2006, 313(5793):1596–1604. 10.1126/science.1128691 16973872

[43] Bailey E, Field L, Rawlings C, King R, Mohareb F, Pak KH, Hughes D, Williamson M, Ganko E, Buer B, et al. A scaffold-level genome assembly of a minute pirate bug, Orius Laevigatus (Hemiptera: Anthocoridae), and a comparative analysis of insecticide resistance-related gene families with hemipteran crop pests. BMC Genomics. 2022;23(1):45. 35012450 10.1186/s12864-021-08249-y PMC8751118

[44] Sparks E, Wachsman G, Benfey PN. Spatiotemporal signalling in plant development. Nat Rev Genet. 2013;14(9):631–44. 23949543 10.1038/nrg3541 PMC3870141

[45] Schoenfelder S, Fraser P. Long-range enhancer-promoter contacts in gene expression control. Nat Rev Genet. 2019;20(8):437–55. 31086298 10.1038/s41576-019-0128-0

[46] Zhang H, Sui Y, Liu W, Yan M, Wang Z, Yan X, Cui H. Identification of a cis-element for long glandular trichome-specific gene expression, which is targeted by a HD-ZIP IV protein. Int J Biol Macromol. 2024;264(Pt 1):130579. 38432280 10.1016/j.ijbiomac.2024.130579

[47] Liu L, Luo S, Ma L, Zhang Y, Wang T, Wang J, Liang X, Xue S. Analysis of ion transport properties of Glycine max HKT transporters and identifying a regulation of GmHKT1;1 by the non-functional GmHKT1;4. *Plant & cell physiology* 2024. 10.1093/pcp/pcae073 38978103

[48] Katebi AR, Kloczkowski A, Jernigan RL. Structural interpretation of protein-protein interaction network. BMC Struct Biol. 2010;10(Suppl 1):S4. 20487511 10.1186/1472-6807-10-S1-S4 PMC2873827

[49] Duby G, Boutry M. The plant plasma membrane proton pump ATPase: a highly regulated P-type ATPase with multiple physiological roles. Pflug Arch: Eur J Physiol. 2009;457(3):645–55. 10.1007/s00424-008-0457-x 18228034

[50] Saddhe AA, Mishra AK, Kumar K. Molecular insights into the role of plant transporters in salt stress response. Physiol Plant. 2021;173(4):1481–94. 33963568 10.1111/ppl.13453

[51] Liu T, Deng S, Zhang C, Yang X, Shi L, Xu F, Wang S, Wang C. Brassinosteroid signaling regulates phosphate starvation-induced malate secretion in plants. J Integr Plant Biol. 2023;65(5):1099–112. 36579777 10.1111/jipb.13443

