# Supplementary figures and images for: Comparative analysis of HKTs in six poplar species and functional characterization of PyHKTs in stress-affected tissues
Source: BMC Genomics. 2025 Jan 7;26:18. doi: 10.1186/s12864-025-11203-x (PMC11708190; doi:10.1186/s12864-025-11203-x)

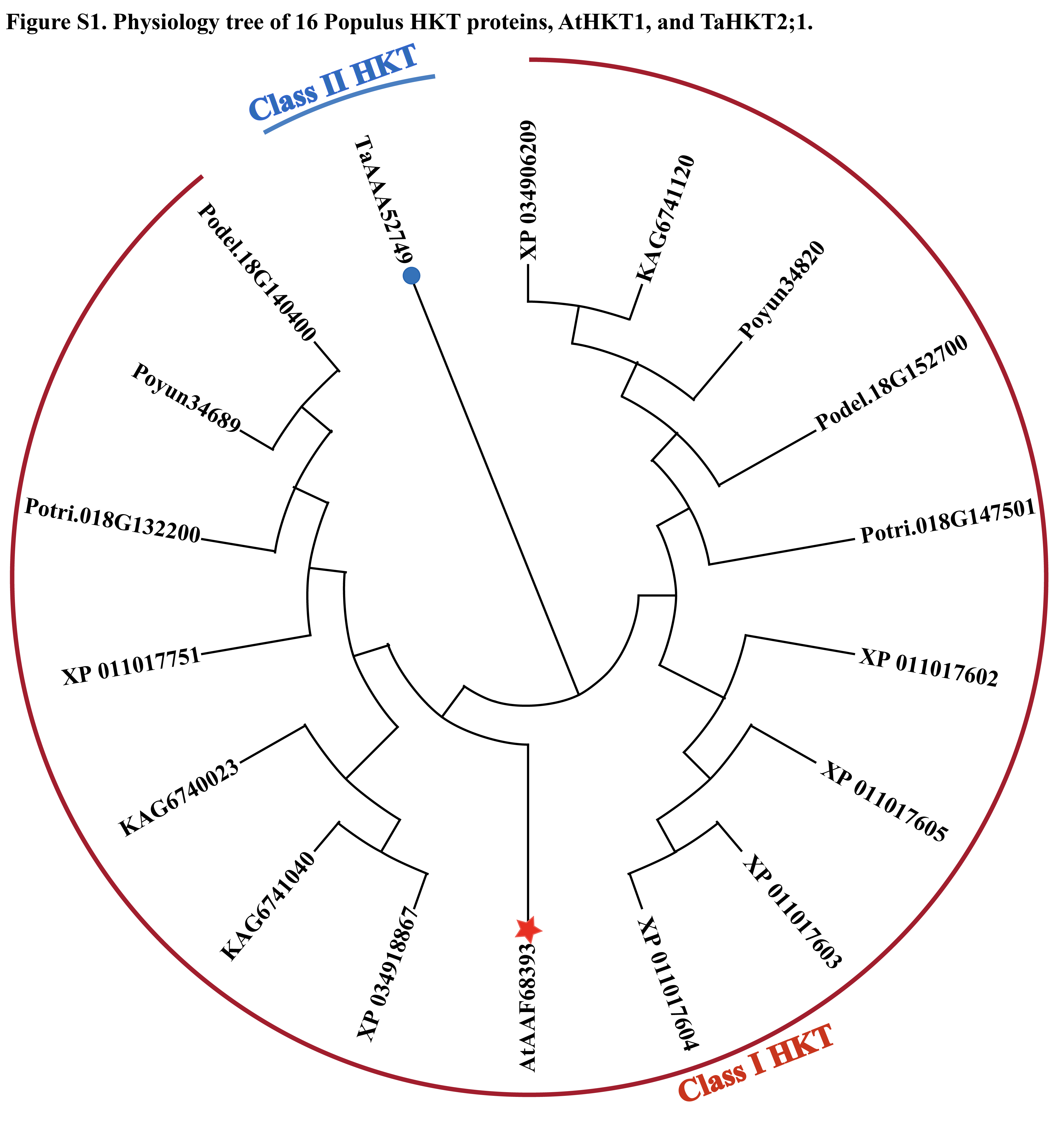

Supplement: Supplementary file 1 — Supplementary Material 1: Physiology tree of 16 Populus HKT proteins, AtHKT1, and TaHKT2;1 [file 12864_2025_11203_MOESM1_ESM.tif]

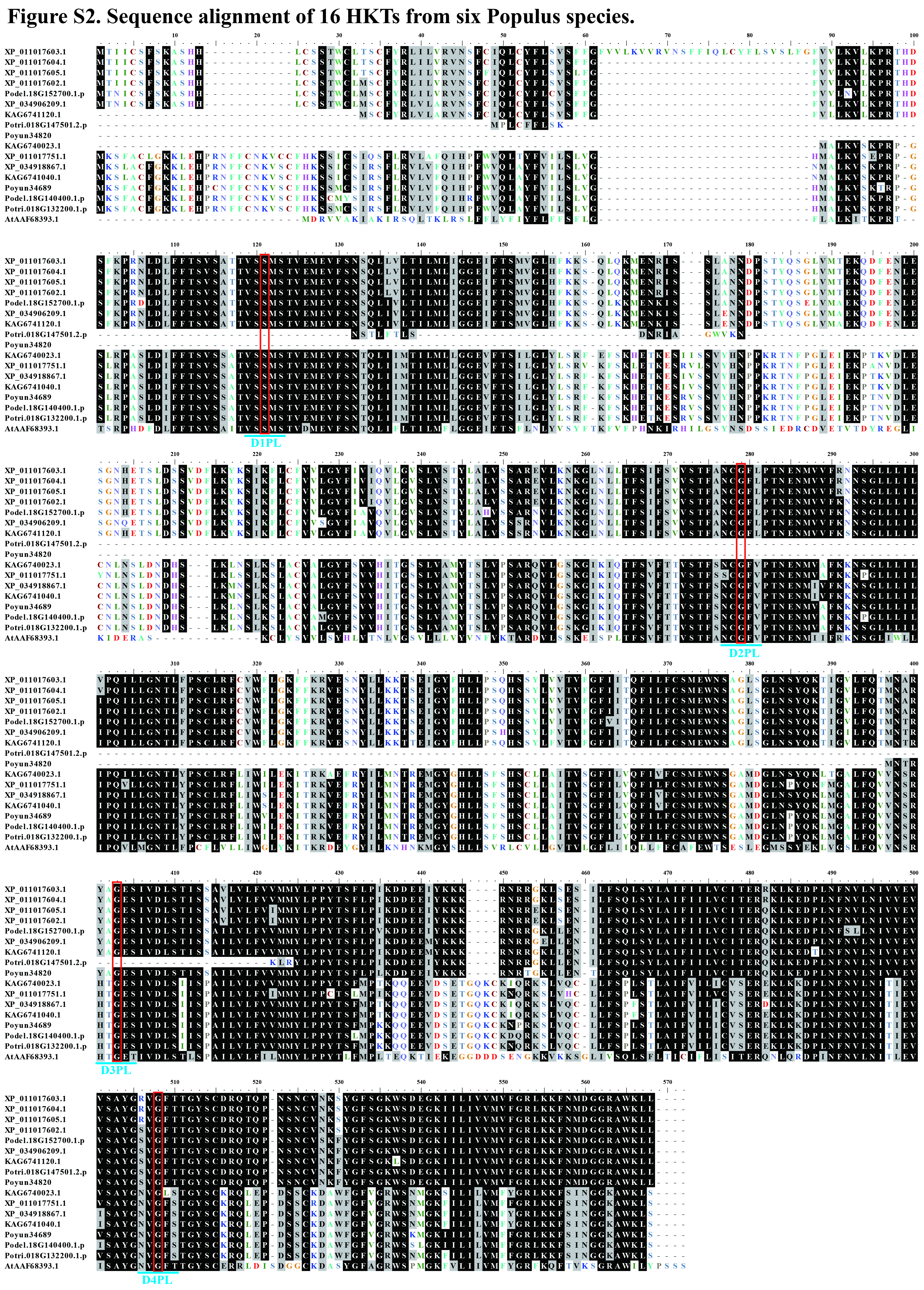

Supplement: Supplementary file 2 — Supplementary Material 2: Sequence alignment of 16 HKTs from six Populus species [file 12864_2025_11203_MOESM2_ESM.tif]

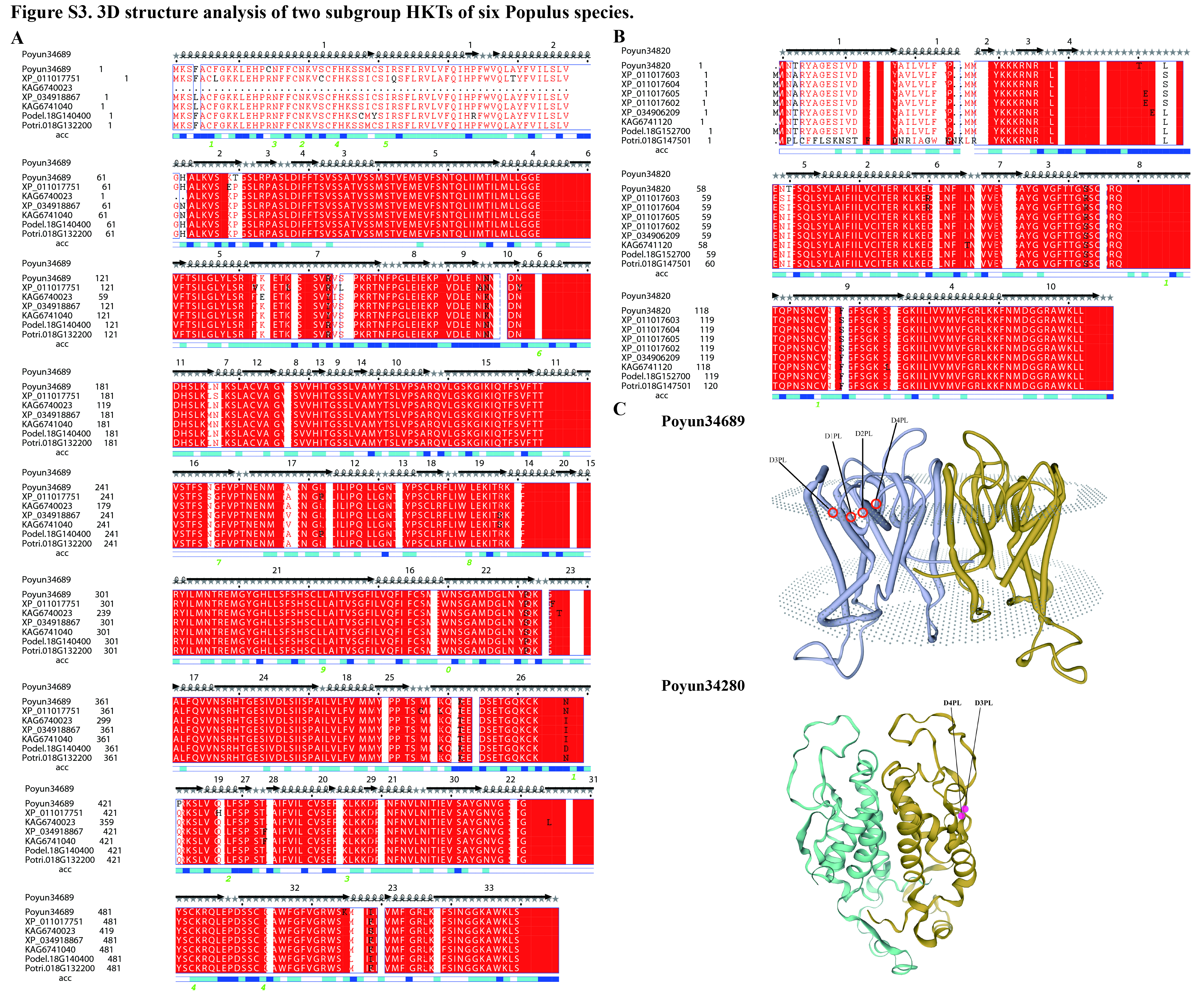

Supplement: Supplementary file 3 — Supplementary Material 3: 3D structure analysis of two subgroup HKTs of six Populus species [file 12864_2025_11203_MOESM3_ESM.tif]

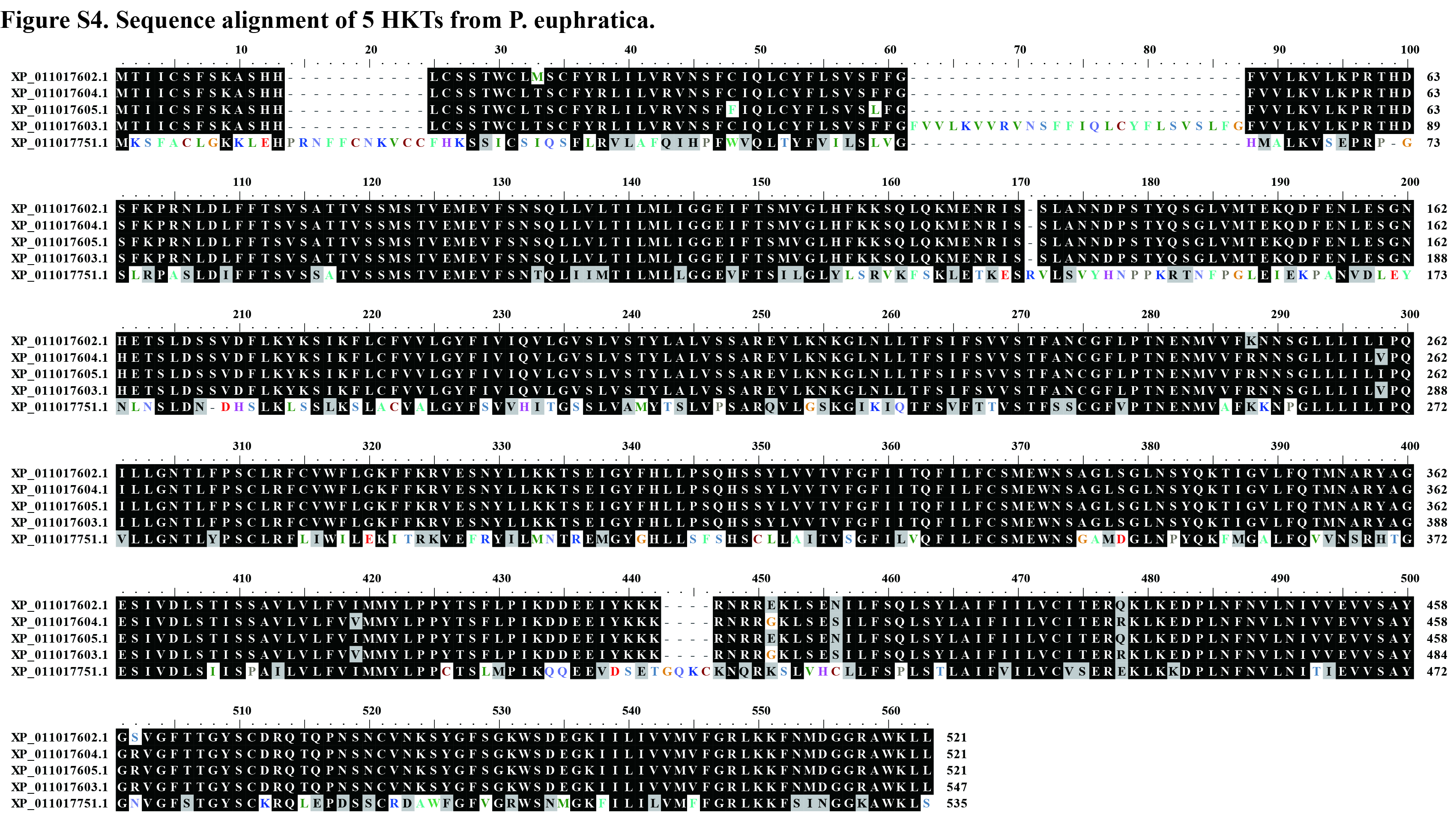

Supplement: Supplementary file 4 — Supplementary Material 4: Sequence alignment of 5 HKTs from P. euphratica [file 12864_2025_11203_MOESM4_ESM.tif]

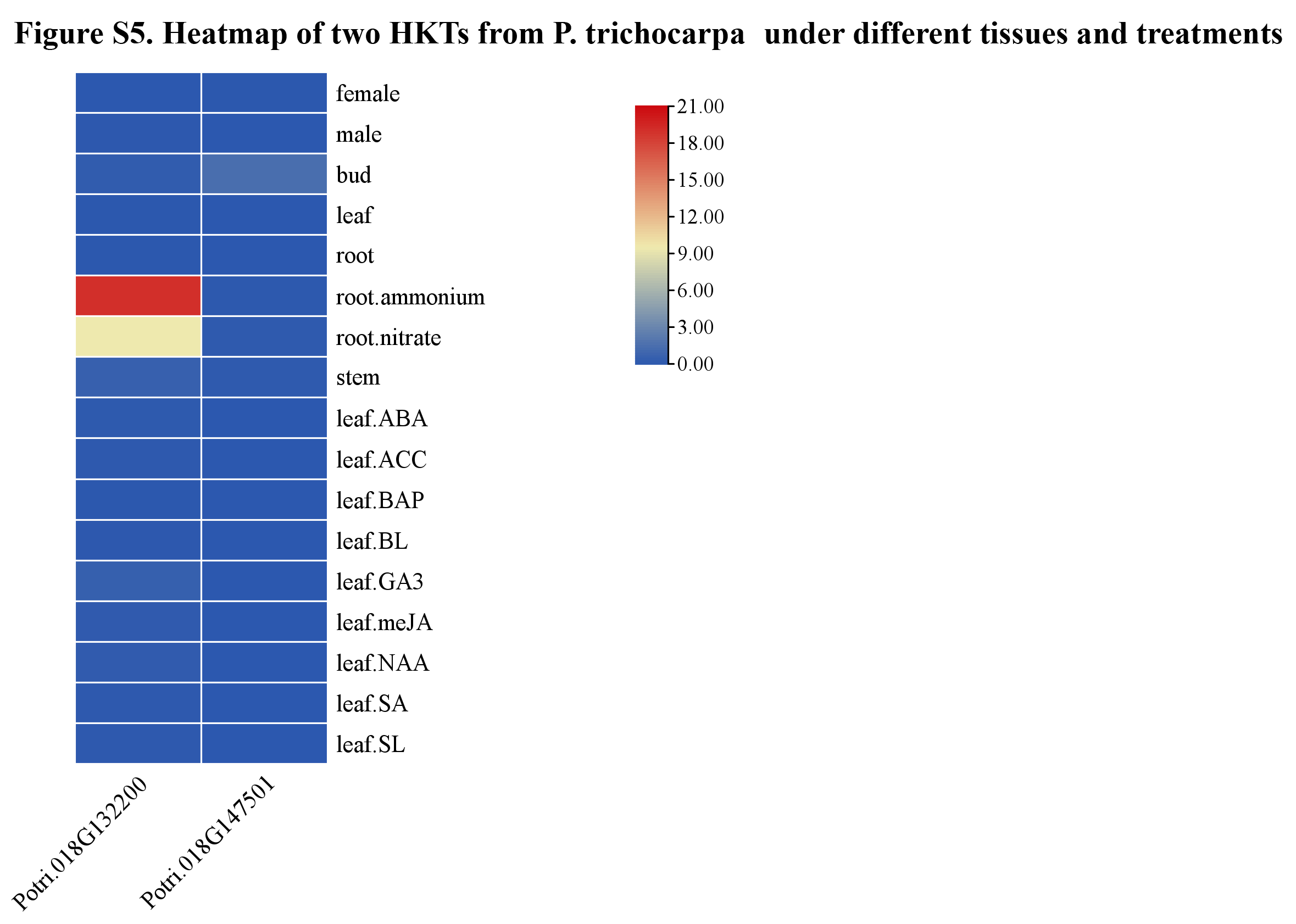

Supplement: Supplementary file 5 — Supplementary Material 5: Heatmap of two HKTs from P. trichocarpa under different tissues and treatments [file 12864_2025_11203_MOESM5_ESM.tif]

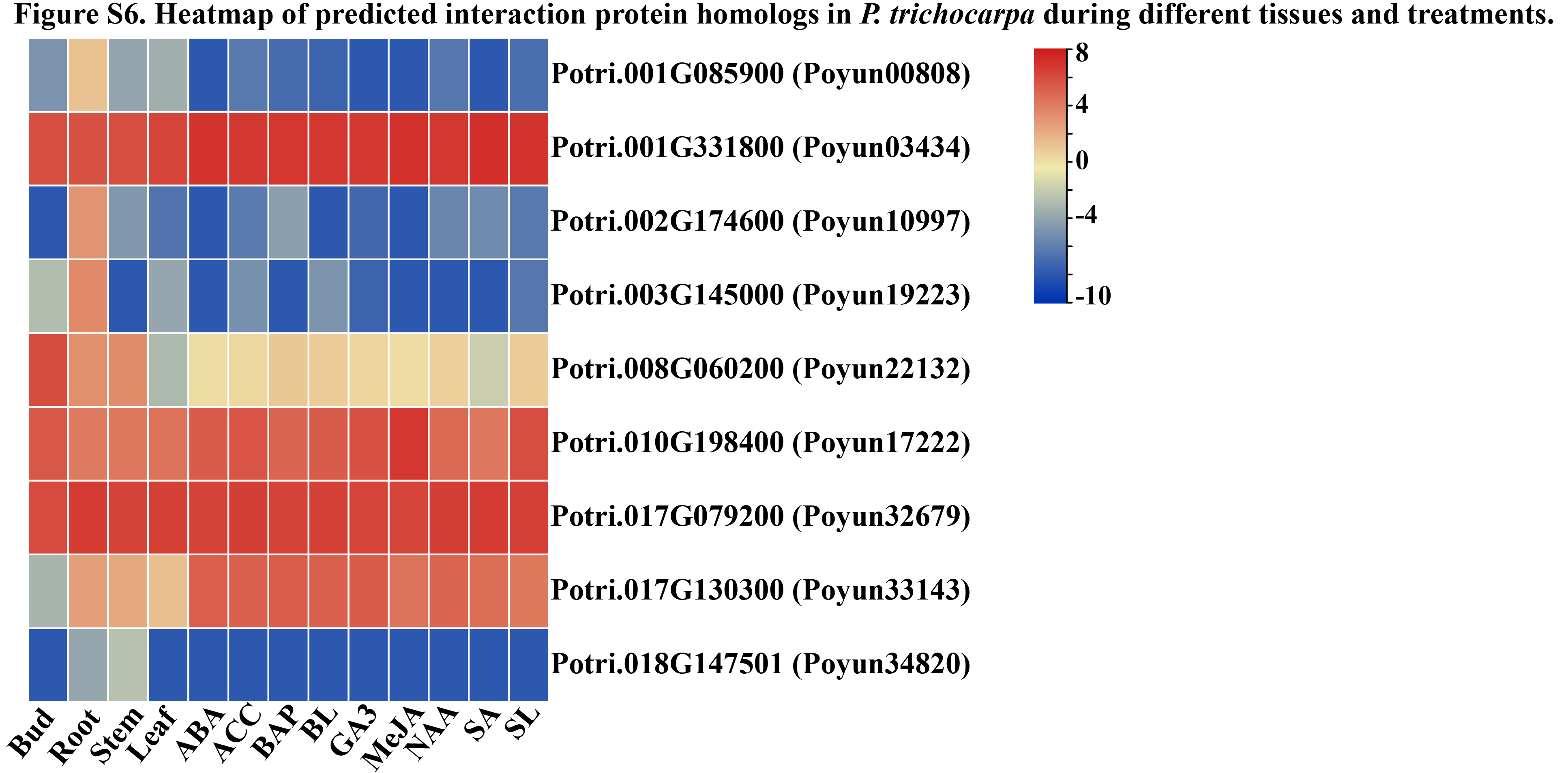

Supplement: Supplementary file 6 — Supplementary Material 6: Heatmap of predicted interaction protein homologs in P. trichocarpa during different tissues and treatments [file 12864_2025_11203_MOESM6_ESM.tif]
